# Comprehensive Immunohistochemical Analysis of Epithelial–Mesenchymal Transition Biomarkers in the Invasive Micropapillary Cancer of the Breast

**DOI:** 10.1155/2024/2350073

**Published:** 2024-06-11

**Authors:** Ozden Oz, Funda Alkan Tasli, Resmiye Irmak Yuzuguldu, Baha Zengel, Demet Kocatepe Cavdar, Merih Guray Durak, Raika Durusoy

**Affiliations:** ^1^ Izmir Bozyaka Training and Research Hospital Department of Pathology University of Health Sciences, Izmir, Türkiye; ^2^ Faculty of Medicine Training and Research Hospital Departments of Pathology Mugla Sıtkı Koçman University, Mugla, Türkiye; ^3^ Medical Faculty Departments of Pathology Dokuz Eylul University, Izmir, Türkiye; ^4^ Department of Public Health Medical Faculty Ege University, Izmir, Türkiye

**Keywords:** biomarker, epithelial–mesenchymal transition (EMT), metastasis, micropapillary breast carcinoma, prognosis

## Abstract

**Background**: Invasive micropapillary carcinoma (IMPC) of the breast is commonly associated with a poor prognosis due to its high incidence of lymphovascular invasion and lymph node metastasis (LNM). Our study is aimed at investigating the prognostic significance of the expressions of E-cadherin (E-cad), N-cadherin (N-cad), CD44s, and *β*-catenin (*β*-cat). In addition, it is aimed at deciphering the consistency of these markers between the IMPC, the invasive breast carcinoma, no-special type (IBC-NST), and LNM components in the same IMPC cases.

**Methods:** Sixty-two IMPC cases with LNM from 1996 to 2018 were analyzed. Immunohistochemical staining was performed separately on the three regions for each patient. Statistical analyses included Kaplan-Meier, Cox regression, and McNemar's statistical tests.

**Results:** Loss of CD44 expression in IMPC, IBC-NST, and LNM areas was associated with poor prognosis in overall survival (OS) (*p* = 0.010, *p* < 0.0005, *p* = 0.025). Loss of CD44 expression in the IBC-NST, gain of N-cad expression in the IMPC, and loss of *β*-cat expression in the LNM areas were indicators of poor prognosis in disease-free survival (DFS) (*p* = 0.005, *p* = 0.041, *p* = 0.009).

**Conclusion:** Our evaluation of this rare subtype, focusing on the expression of key epithelial–mesenchymal transition (EMT) molecules, revealed that it shares characteristics with the IBC-NST component within mixed tumors. Notably, contrary to expectations, a reduction in CD44 expression was found to adversely affect both OS and DFS. By conducting staining procedures simultaneously across three regions within the same patient, a novel approach has provided valuable insights into the mechanisms of EMT.

## 1. Introduction

Metastasis and recurrence rates in breast carcinomas surpass those observed in many other cancer types, posing a significant burden in terms of morbidity and mortality [1–3]. One particularly rare subtype among breast carcinomas is invasive micropapillary carcinoma (IMPC), with an incidence ranging from 0.9% to 2.6% of all breast cancers [4–6]. Initially characterized by Fisher et al. in 1980 as having an “exfoliative appearance” [7], this distinctive morphological pattern was subsequently termed “invasive micropapillary carcinoma of the breast” by Siriaunkgul and Tavassoli, eventually being recognized in tumors originating from various organs, including the bladder [8], colon [9], lung [10], and even salivary gland [11]. The heightened interest in IMPC since the early 2000s has stemmed from its association with a higher frequency of lymphovascular invasion (LVI) and LNM compared to other breast carcinoma subtypes [6, 12–14]. It has been generally reported that IMPC is characterized by an unfavorable prognosis [6, 7, 15–18].

**Figure 1:**
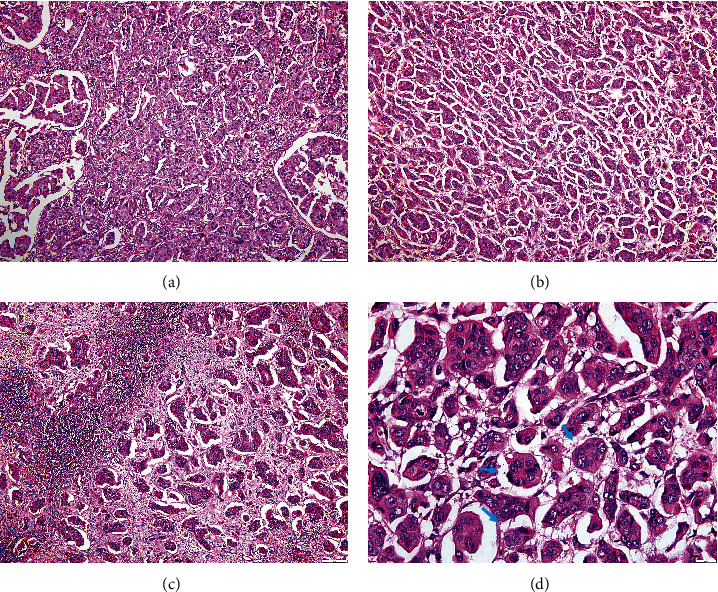
Examples of H&E staining of invasive micropapillary carcinoma (IMPC) cases: (a, b, d) Examples from the primary IMPC areas. (c) Example from areas of IMPC metastasis in the lymph node. (d) Blue arrows indicate groups of morule-like cells without papillary core and separated from the stroma ((a–c) ×10 and (d) ×40 magnifications).

IMPC has a distinctive histopathological presentation. Small clusters of tumor cells are notably devoid of a fibrovascular core or stromal tissue [5, 7, 17] (Figure 1). The identification of the “inside out” staining pattern through EMA immunohistochemical (IHC) analysis on these morule-like clusters is important supportive evidence [17].

The existing genetic studies present conflicting viewpoints regarding IMPC in breast cancer. While some support the notion that IMPCs possess distinct genetic profiles, suggesting it as a distinct pathological variant [19], some have failed to pinpoint specific genomic aberrations accounting for IMPC's unique morphology and clinical behavior [7].

The existing prognostic studies present conflicting viewpoints regarding IMPC, too. While earlier studies generally assert that patients exhibit lower 5-year and 10-year survival rates [4, 6], recent investigations propose that recurrence and survival rates in IMPC are comparable to those in IBC-NST. Recent studies also contend that aggressive clinical interventions may not be warranted in IMPC cases [7, 12, 14, 16, 20, 21]. Previous studies also argue that any proportion of IMPC component is a significant predictive factor for LNM [4, 12, 13]. Despite the absence of consensus in the literature regarding IMPC's prognostic data, it remains categorized as having a poor prognosis in the 2019 WHO classification [5].

Epithelial–mesenchymal transition (EMT) is a pivotal process activated during cancer invasion, and IMPC is a good candidate subtype for a comprehensive investigation of the EMT. It is widely acknowledged that during EMT, there is a reduction in the synthesis of molecules associated with epithelial characteristics and an increase in molecules linked to mesenchymal attributes [22–25].

Although the identification of certain molecular markers in cancer cells undergoing EMT remains unclear, the prominent acknowledgment is that the substitution of E-cadherin (E-cad) with N-cadherin (N-cad) is a fundamental molecular hallmark of this process [26, 27].

**Figure 2:**
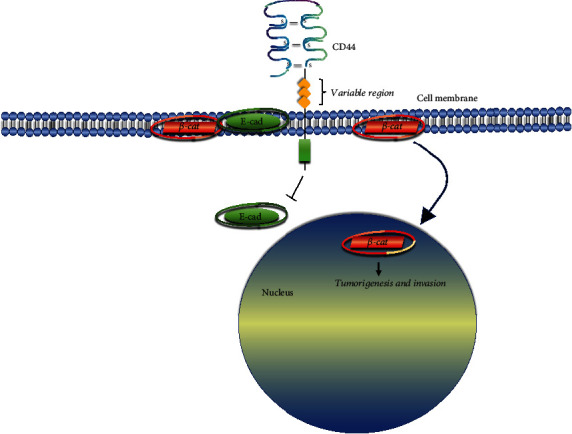
Representative signaling pathway induced by CD44, E-cadherin, and *β*-catenin relationship: CD44 separates the membrane-associated E-cadherin and *β*-catenin complex and inhibits E-cadherin. The liberated *β*-catenin migrates from the membrane to the nucleus, and then, it activates the cell invasion genes involved in tumorigenesis [33, 34].

It has been posited that the transition from isoform variants (CD44v) of the CD44 protein, a recognized coreceptor involved in the regulation of cellular signaling pathways during the EMT process, to the standard isoform (CD44s) contributes significantly to this phenomenon [28, 29]. CD44 exerts its influence on *β*-catenin (*β*-cat), a protein residing on the cell membrane in conjunction with E-cad. CD44 suppresses the expression of E-cad, disrupting the colocalization of E-cad and *β*-cat at the cell membrane. Consequently, liberated *β*-cat translocates to the cytoplasm and nucleus, where it activates the synthesis of genes associated with the EMT process (Figure 2) [30–34].

In our research, we delve into the quantification of expression levels of well-defined molecules associated with the EMT process within IMPC tumor cells. To discern disparities in expression, we simultaneously and independently assessed the IMPC region, the IBC-NST region in mixed tumors, and the region corresponding to LNM in the same patient. This distinctive approach serves as the foundational framework of our investigation. Within this framework, initially, we investigated the correlation between alterations in molecular expression in three regions and various clinical outcomes, including overall survival (OS), disease-free survival (DFS), and other pertinent prognostic parameters. Moreover, we compared the immunohistochemistry results of these molecules in the tumoral region with the LNM region in the same patient to discuss their interrelationships.

## 2. Material and Methods

### 2.1. Patient Population

#### 2.1.1. Clinicopathological Data

In this retrospective cohort investigation, we conducted an in-depth analysis of 2,916 breast cancer cases diagnosed between 1996 and 2018 at the DEUMF and UHSIBTRH Pathology Departments. This study was conducted in compliance with the local ethics committee's approval (study no. = 2021/16, date = January 26, 2021). Among this cohort, we specifically focused on 149 cases diagnosed with IMPC. We meticulously excluded 87 cases from our analysis for various reasons, such as missing clinical follow-up data, receiving neoadjuvant treatment, consultation cases, micrometastasis, and no LNM. Ultimately, our study included 62 cases. The digital archive systems of both hospitals were used to obtain clinical data.

#### 2.1.2. Histopathologic Examination

In this study, a comprehensive histopathological re-evaluation of 62 archived cases was conducted by three pathologists, who meticulously examined H&E and IHC stained (molecular classification) sections and documented their histopathological observations. Numerical prognostic parameters, including patient age, tumor size (TS), presence of a second tumor focus, extent of intraductal components, LNM, OS, and DFS, were meticulously recorded. Additionally, we categorized prognostic factors such as perineural invasion (PNI), lymphatic invasion (LI), vascular invasion (VI), the occurrence of local or distant organ recurrence, skin involvement, and the presence of macroscopic or microscopic tumor involvement at the resection margin. Furthermore, detailed data were collected on the type of surgical procedures performed, and the clinical and pathological staging, classified according to the 5th edition of the World Health Organization's Tumor Classification of Breast Tumors [5].

#### 2.1.3. Survival Data

In this study, survival data was extracted from the Local Cancer Monitoring archive records, taking into account patient follow-up information. We calculated the OS as the time elapsed between the date of the initial Tru-cut biopsy diagnosis and either the date of death or the last recorded observation time for patients still living. Additionally, for both living and deceased patients, we computed the DFS by measuring the interval between the date of the initial Tru-cut biopsy diagnosis and the recurrence event. It is important to note that our dataset's follow-up information was last updated in August 2021.

### 2.2. Tissue Microarrays (TMAs)

In our cohort study, we carefully selected three distinct regions within mixed IMPC tumors, utilizing FFPE primary tumor tissues. These regions of interest comprised the IMPC component, the IBC-NST component, and the LNM, with a preference for the IMPC component when available. For clarity, we denoted these regions as “I” for IMPC, “II” for IBC-NST, and “III” for LNM in individual patient specimens. In the case of pure IMPC tumors, we selected only two regions for analysis: the IMPC and LNM. The generation of TMAs was carried out following the established protocol described by Kononen et al. [35].

Prior to conducting our analyses, approval was obtained from the local ethics committee to utilize and assess archived patient tissue blocks, adhering to the principles outlined in the Helsinki Declaration.

### 2.3. Immunohistochemistry

Molecular classification data were reanalyzed from the pathology slide archive. To investigate the phenomenon of EMT, we conducted IHC staining on a TMA. Each microarray block yielded 4-micron-thick sections for IHC analysis. These sections underwent a 5-min heat-induced antigen retrieval process in a pressure cooker at 121°C, using pH 9 DakoTarget Retrieval Solution™ (Agilent, CA, USA; #S2367). Table S1 provides detailed information regarding the antibodies employed in our study. We employed the Dako DAB detection kit on a Dako autostainer to visualize antibody binding.

#### 2.3.1. Interpretation of IHC Staining

In accordance with the antibody characteristics, we employed positive control tissues for various markers: normal small intestine tissue for CD44, normal tonsillar tissue for E-cad, normal appendix tissue for *β*-cat, and normal liver tissue for N-cad. Notably, we considered cytoplasmic and/or membranous staining patterns as indicators of positive protein expression for CD44, E-cad, N-cad, and *β*-cat.

##### 2.3.1.1. IHC Staining for Molecular Classification

In our study, we assessed the expression of ER and PR by analyzing nuclear-staining positivity. We quantified the proportion of positively stained cells as a percentage of the entire stained area. We evaluated the expression of Ki-67 by calculating the percentage of cells with nuclear staining among a sample of 100 tumor cells in the most intensely stained region. Lastly, we evaluated the expression of Her-2-neu according to the 2018 ASCO/CAP guidelines [36]. Tumors exhibiting 3+ complete membranous staining in more than 10% of cells were categorized as positive for Cerb-B2 (Her-2-neu).

##### 2.3.1.2. Molecular Classification

In our research cohort, we adopted the molecular classification criteria established by the 2011 St. Gallen consensus to delineate five distinct subtypes of breast cancer [37].

##### 2.3.1.3. Immunostaining for EMT Molecules

The IHC for CD44, *β*-cat, N-cad, and E-cad involved a dual evaluation based on the intensity and the proportion of cell staining. We utilized the “The immune reactive scoring (IRS)” system. Consequently, we ranked the scores ranging from 0 to 3, with 0 representing a negative score, 1 indicating *weak staining*, 2 denoting *moderate staining*, and 3 signifying *strong staining*.

Importantly, all assessments were conducted by pathologists blinded to any pathological or clinical information, ensuring unbiased evaluation.

We grouped the CD44 staining that scored ≤ 6 as negative and ≥ 7 as positive and *β*-cat and N-cad staining that scored ≤ 3 as negative and ≥ 4 as positive to ensure a balanced distribution of cases in prognostic statistical studies.

### 2.4. Statistical Analysis

We performed a comprehensive statistical analysis to investigate the associations between prognostic parameters and biomarkers using IBM SPSS® software version 18.0 (Statistical Packages for the Social Sciences, Chicago, IL). Categorical variables were compared using the Chi-square test. To assess differences among the groups, we utilized the Mann–Whitney *U* test for numeric features, while age comparisons were conducted with Student's *t*-test. We employed the Kaplan-Meier analysis to compare survival outcomes across various groups for univariate survival analyses.

We interrogated independent prognostic features using the Cox regression survival analysis. To assess differences in staining patterns between I and II, I and III, and II and III, McNemar's test was utilized.

## 3. Results

### 3.1. Cohort Group Characteristics

In all IMPC cases, we observed a 62.63% rate of LNM, excluding those with micrometastases. The median age of the patients was 57.50 years, ranging from 31 to 82, with a mean age of 55.45 ± 12.00 years in the cohort. The mean OS time was 91.92 ± 56.09 months. Notably, patients aged 55 and above had an expected life expectancy of 116.144 ± 13.905 months, whereas those below 55 exhibited an expected life expectancy of 184.004 ± 18.978 months (*p* = 0.05).

Our analysis of survival outcomes revealed that the cumulative proportion of patients surviving at the 12th, 24th, 48th, 60th, and 120th months was 93%, 89%, 82%, 76%, and 59%, respectively. The median follow-up duration for our cohort was 87.50 ± 56.40 months (2–250).

Regarding tumor characteristics, the median TS was 22.00 mm (7–100) and a mean size of 28.88 ± 19.35 mm. Half of the tumors measured below 22 mm, and 75% were smaller than 33 mm. As expected, patients with tumors smaller than 20 mm exhibited significantly longer OS and distant metastasis-free survival expectations (*p* < 0.0005 and *p* = 0.001, respectively). Furthermore, we noted the presence of a second tumor focus in 27.4% of the cases, apart from the primary mass.

### 3.2. Clinicopathological Descriptive Parameters

In eight (12.9%) instances, regions exhibiting micropapillary patterns were exclusively observed within the entire cancer mass, categorizing them as pure IMPCs. In the remaining cases, various proportions of the micropapillary pattern were detected. Of the patients in the study, 29 exhibited right-sided, 28 left-sided, and 5 bilateral breast cancer. Tumor localization was primarily in the UOQ in 30 cases (48.4%), multifocal in 12 cases (19.4%), LOQ in 10 patients (16.1%), and U&LIQ in 10 cases (16.1%).

49.2% of the number of LNM presented with ≤ 2. Remarkably, 28% of cases with LNM had ≥ 6 lymph node metastases. The mean number of LNM was 5.85, with a median of 3 (1–46). The mean size of the largest metastatic lymph node was 17.04 ± 8.11 mm, with 50% measuring 15 mm or less and 75% being smaller than 20 mm. Pericapsular invasion was detected in 59.7% of cases, and perinodular adipose tissue spread was observed in 13.9% of metastatic lymph nodes. During follow-up, local recurrence was identified in three patients (4.8%), while distant organ metastases occurred in 18 patients (29.1%), primarily affecting the lungs (61%), with other sites, including the bone, liver, and extra-axillary lymph nodes. Encouragingly, 41 patients (66.1%) did not experience any recurrence.

Breast skin involvement was documented in 15 patients (24.2%). Among the cases, 38.7% were classified under the pathological stage of pT1cN3M0, while the remainder exhibited higher stages. Clinical staging indicated that 54.8% of patients fell within stage ≤ 2B. The Bloom-Richardson score revealed that 35 cases (56.4%) scored three. Positive surgical margins were detected in 9.7% of the cases (six cases) with breast-conserving surgery. All these patients underwent surgical cavity revision and additional radiation therapy. VI was identified in 36 cases (58.1%), while PNI was observed in 10 cases (16.1%). Furthermore, LI was noted in 49 cases (79%). Surgical management varied, with 30 cases (48.4%) undergoing breast-conserving surgery in combination with sentinel lymph node biopsy or axillary dissection and the remaining 32 cases (51.6%) undergoing modified mastectomy with the same lymph node evaluation procedures.

### 3.3. OS and DFS

#### 3.3.1. The Histopathologic Features

In our cohort study, we observed a noteworthy difference in total survival time among patients with tumors located in the UIQ and LOQ. In our cases with pure IMPC, when compared to the mixed IMPC group, there was no statistically significant distinction in OS between them (*p* = 0.636).

**Figure 3:**
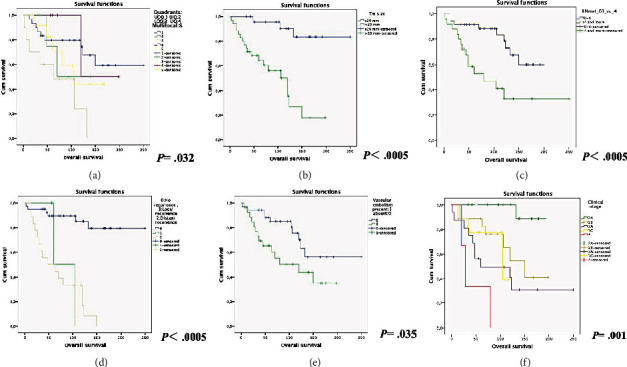
The histopathological parameters affecting prognosis in invasive micropapillary carcinoma (IMPC) and their Kaplan-Meier plots: (a) LOQ (lower outer quadrant) is associated with adverse prognosis in IMPC (*p* = 0.032). (b) tumor size (> 20 mm) is associated with adverse prognosis in IMPC (*p* < 0.0005). (c) Lymph node metastasis (≧ 4) is associated with adverse prognosis in IMPC (*p* < 0.0005). (d) Distant recurrence is associated with adverse prognosis in IMPC (*p* < 0.0005). (e) Vascular invasion is associated with adverse prognosis in IMPC (*p* = 0.035). (f) Clinical stage is associated with adverse prognosis in IMPC (*p* = 0.001).

Several key factors significantly influenced the OS of our patients. TS (≦ 2 cm), LNM, (< 4), no recurrence, negative VI, and patients with clinical stage 2B or lower were all linked to longer life expectancies. Comprehensive information regarding the factors impacting the OS of our patients and their expected survival is presented with the Kaplan-Meier survival curves illustrated in Figure 3.

### 3.4. IHC Molecular Features

#### 3.4.1. IHC Results of Molecular Classification

##### 3.4.1.1. ER/PR/Her2/Ki67 Immunostaining

Out of 62 cases, 53 (85.51%) were ER+, 46 (74.19%) were PR+, and 27 (43.54%) were Her‐2 + (15 scor : 2 (FISH+)/12 scor : 3). The mean Ki67 rate was 37.88 ± 25.18% (5%–90%).

##### 3.4.1.2. Molecular Classification Results

Eleven cases (17.7%) were classified as luminal A, 22 cases (35.5%) as luminal B, 22 cases (35.5%) as luminal B and Her-2, 5 cases (8.1%) as Her-2 rich, and 2 cases (03.2%) as triple negative.

#### 3.4.2. Immunostaining Results With EMT Biomarkers and OS-DFS

##### 3.4.2.1. CD44 Immunostaining

**Figure 4:**
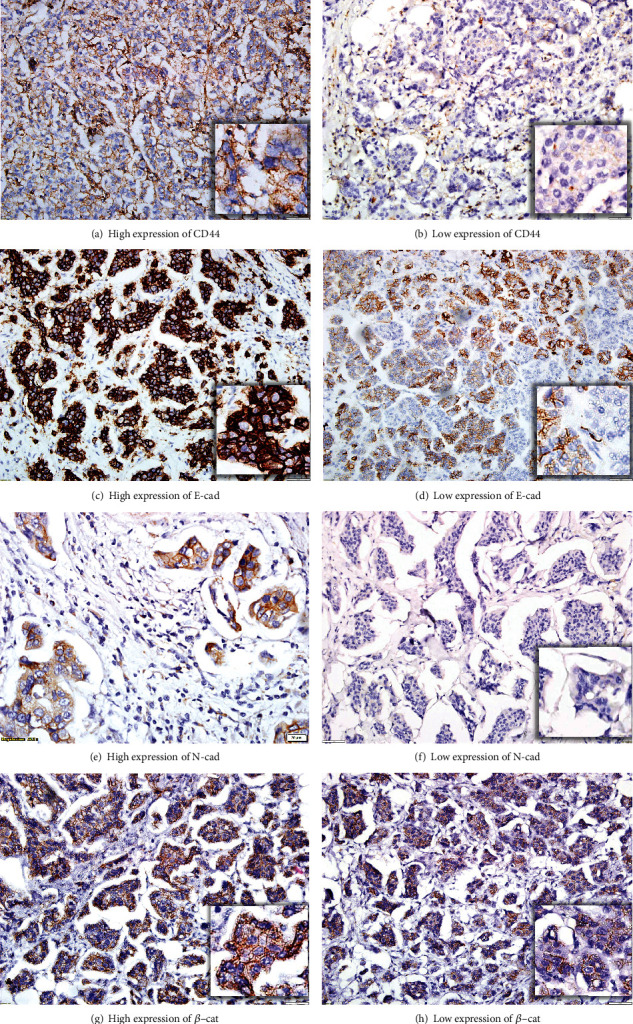
The immunohistochemical staining results and scoring samples with CD44, E-cadherin (E-cad), N-cadherin (N-cad), and *β*-catenin (*β*-cat): The staining scorings were done by considering only membranous staining differences of tumor cells. (a, b) Examples of CD44 staining intensity with high and low intensity. (c, d) Examples of E-cad staining scores with high and low intensity. (e, f) Examples of N-cad staining scores with high and low intensity. (g, h) Examples of *β*-cat staining scores with high and low intensity ((a–d, f–h) images ×20 and (e) small images, ×40 magnification).

We observed a significant correlation between CD44 expression levels and patient outcomes in our study. Specifically, we found that low or absent CD44 expression (scores 0–6) in regions characterized as IMPC (I), IBC-NST (II), and LNM (III) was associated with shorter OS expectancy (Table 1). Furthermore, we observed that the loss of CD44 expression in the context of IBC-NST(II) within mixed tumors was associated with a shortened DFS time (Table 1). Illustrative samples of CD44 IHC staining scoring from tumor tissues are presented in Figure 4.

##### 3.4.2.2. *β*-cat Immunostaining

We found a notable reduction in the membranous expression of *β*-cat in LNM (III), specifically observed in 10 cases. This decrease in *β*-cat expression was associated with significantly shortening the DFS time (Table 1). Figure 4 illustrates representative samples of tumor tissues used for *β*-cat IHC staining scoring.

##### 3.4.2.3. N-cad Immunostaining

We observed moderate to severe membrane positivity (scores 4–9) for N-cad expression within the IMPC areas in 29 cases. Our analysis revealed that individuals with these higher N-cad scores (> 3) exhibited significantly reduced DFS, as demonstrated in Table 1. For a visual representation of N-cad staining in tumor tissues, please refer to Figure 4.

##### 3.4.2.4. E-cad Immunostaining

There was no statistical difference between scoring with the E-cad biomarker. Samples obtained from tumor tissues of the cases for E-cad are demonstrated in Figure 4.

#### 3.4.3. Comparison of Results of Prognostic Significance IHC Staining With the Other Prognostic Parameters (Cox Regression Test Results)

In the initial phase of our investigation, we examined the correlations among CD44 I and CD44 II staining outcomes in tumor tissue, which we observed to influence OS potentially with TS and LVI. Our analysis revealed that when CD44 I and TS were jointly assessed, they did not demonstrate independent prognostic significance (*p* = 0.079 and *p* = 0.001, respectively). However, when CD44 II and TS were considered together, they emerged as independent prognostic factors (*p* = 0.035 and *p* = 0.001, respectively), signifying their potential impact on patient outcomes. No independent associations were identified between these staining and other clinical parameters.

Furthermore, we extended our investigation to evaluate the impact of CD44 II, *β*-cat III, and N-cad I stainings in conjunction with TS and LNM on patient DFS using the Cox regression survival analysis. Regrettably, no independent prognostic effects were observed for these staining results, indicating their limited influence on DFS.

#### 3.4.4. The Comparison of Immunostaining Results of EMT Biomarkers in IMPC (I), IBC-NST (II), and LNM (III)

Differential expression patterns of *β*-cat, N-cad, CD44, and E-cad were observed within tissue samples obtained from the same patient across three distinct regions. The statistical analysis revealed highly significant disparities in the staining rates of *β*-cat and N-cad across all three regions (*p* < 0.000, *p* < 0.000, *p* < 0.000, respectively). Furthermore, significant differences were detected in the staining rates of *β*-cat and CD44 within all three regions (*p* = 0.021, *p* = 0.005, *p* = 0.012, respectively). Interestingly, while no significant differences were noted between *β*-cat and E-cad staining rates in the I and II regions, a significant disparity was observed in the III region (*p* = 0.180, *p* = 0.167, *p* = 0.007, respectively).

In contrast, N-cad and CD44 staining rates showed no significant differences in the I and III regions but exhibited a significant difference in the II region (*p* = 0.015, *p* = 0.233, *p* = 0.001, respectively). Moreover, differences in staining rates were identified between N-cad and E-cad in all three regions (*p* < 0.000, *p* < 0.000, and *p* < 0.000, respectively). Notably, there were no statistically significant differences between CD44 and E-cad staining rates across all three regions (*p* < 0.345, *p* < 0.980, and *p* < 0.701, respectively) (Table 2).

## 4. Discussion

Early assessment of the potential for metastasis in breast carcinoma, prior to its metastatic status, can significantly impact treatment criteria. The intricate molecular mechanisms governing the capacity for “invasion and metastasis,” a pivotal hallmark of cancer, remain incompletely understood [22–25].

When considering the existing body of literature, it is widely acknowledged that IMPC is a subtype of breast carcinoma characterized by a heightened propensity for metastasis [4, 6, 14–18, 38]. Our study encompassed 62 patients who exhibited LNM out of 99 who did not undergo neoadjuvant chemotherapy. Our vascular embolism rate is 41.90%, a finding consistent with previous research [4, 14, 39]. Importantly, the vascular embolism rate in IMPC was higher than that observed in cases of IBC-NST [12, 14]. Our analysis revealed significant associations between the number of LNM, TS, recurrence, clinical stage, and prognosis (chart). These descriptive findings remain congruent with the existing literature and exhibit similarities to IBC-NST cases [6, 12, 14, 16].

In our study, among the 62 cases studied, 55 of them (88.70%) exhibited ER positivity. Our molecular classification results align with previous findings in the literature [12, 14, 16, 19, 21]. Notably, the luminal B subtype prevalence in the IMPC group reached 71.00%, indicating a higher proliferation index than IBC-NST [20]. We identified 27 cases as Her-2 positive (43.54%), consistent with reported rates in the literature [4, 39], although some studies have reported lower percentages [16, 18, 19, 21]. Additionally, the prevalence of the Her-2-rich subclass at 8.1% aligns with previous findings in the literature [16, 39]. In contrast, our case group exhibited a low incidence of triple-negative cases (3.2%), which is consistent with documented rates in the literature [16, 18].

First, we will discuss our staining results and their prognostic implications. Subsequently, we will analyze and discuss the concordance and disparities observed among the results obtained from these three distinct regions (IMPC (I), IBC-NST (II), and LNM (III)). This approach sheds light on the molecular mechanisms underlying EMT in breast cancer and provides valuable insights into the clinical relevance of our findings. It is important to note that no previous research with a similar methodology was conducted in the literature.

CD44 is a transmembrane glycoprotein with multiple posttranslational modifications. It influences cellular signaling by engaging in coreceptor complexes with various receptor tyrosine kinases. CD44 interacts with various extracellular matrix (ECM) ligands, most notably hyaluronic acid [2, 3, 34]. Alterations in the ECM can shift CD44 expression from its variant isoform (CD44v) to the standard isoform (CD44s) and result in overexpression of N-cad. This transformation has been documented particularly in high-grade breast carcinoma [28]. Contrarily, it is contended that CD44s exhibit significant expression in most tissues, while CD44 variants are predominantly expressed in tumor cells [3]. Our research group also investigated the same CD44s clone utilized by Badyal et al., and our findings align with theirs [40]. Although it has long been suggested that CD44v6 plays a role in breast cancer, their underlying mechanisms remain incompletely understood [3, 28, 29, 34]. In a study assessing the localization of CD44v5 and v9 exclusively on the apical membrane in IMPC, it was observed that their expression decreased, although the difference from IBC-NST was not statistically significant [15]. Some investigations have reported reduced expression of CD44s, CD44v6, and CD44v9 in IMPC [41]. Given that micropapillary clusters exhibit strong intercellular adhesion and minimal adhesion to the surrounding tissue, it is conceivable that the loss of CD44 as an adhesion molecule in these clusters is a natural process. Consequently, when evaluating our CD44 staining results, we not only assessed CD44 staining on the apical surface of morules, as in the studies by Simonetti et al., but also examined the loss of total CD44 membranous expression within tumoral cell morules. Another reason for evaluating complete CD44 staining in morules was the necessity to apply the same assessments to the second focus in mixed tumors and the third focus in the LNM for comparative purposes. This approach is equally applicable to other markers. It was thought that the inconsistencies in the literature resulted from different approaches when evaluating the staining results. CD44 exerts inhibitory effects on E-cad expression, disrupting their colocalization in the cell membrane. Consequently, *β*-cat is released into the cytoplasm and nucleus, where it activates the synthesis of genes associated with EMT, ultimately leading to increased expression of A-SMA, N-cad, and Vim [33, 34] (Figure 2). The role of CD44 in tumorigenesis remains a topic of conflicting data in the literature [42]. In our cohort series, we have demonstrated that the loss of CD44 expression in areas I, II, and III is associated with poor prognostic outcomes in terms of OS (Table 1). These findings suggest that the loss of CD44 as an adhesion molecule serves as an adverse prognostic parameter [40, 41]. Our study contradicts the observations of Umeda et al., who reported a differential loss of CD44s expression between the IMPC and IBC-NST areas in a study involving 31 mixed IMPC cases [41]. On the contrary, we found no significant difference in CD44 expression loss between the IMPC and IBC-NST areas, in line with the work of Gong et al. comparing IMPC and tubular carcinoma [43]. Moreover, we have demonstrated that the loss of CD44 expression exclusively in the IBC-NST areas is also linked to poorer outcomes in terms of DFS (Table 1), which is inconsistent with the prevailing notion that the IMPC component drives metastasis in mixed cases [6, 12–14, 40, 44]. Thus, our findings suggest that the loss of CD44 expression may contribute more significantly to LVI and metastasis in IBC-NST than in the IMPC component. Moreover, our comprehensive analysis of CD44 II staining demonstrated its independent prognostic effects in our study.

We did not observe a complete absence of E-cad expression; instead, we detected only a minimal reduction in its expression levels. Notably, the expected decline in E-cad expression typically observed in invasive lobular and basaloid-type breast carcinomas was not statistically significant within our IMPC cohort. These results indirectly lend support to the observation of a low incidence of the triple-negative molecular subtype of breast cancer (3.2%) within our study group.

Following our investigation into the N-cad biomarker, we have made a noteworthy discovery that aligns with EMT mechanisms. Specifically, we have observed a shorter DFS in cases exhibiting increased N-cad expression within the IMPC region (Table 1). It is important to note that research in this area is limited, and our findings regarding N-cad are in line with the study conducted by Nagi et al. [27]. These findings lend support to existing literature suggesting that the IMPC component possesses greater metastatic potential in terms of N-cad expression, which is anticipated to increase in metastatic cells [6, 7, 15–18].

In our study, the loss of CD44 expression in the IBC-NST group and the gain of N-cad expression in the IMPC component were associated with an increased metastatic potential. These results lead us to interpret that different metastatic pathways are at play within the two regions. It is worth noting that our discovery of increased N-cad expression does not impact OS, consistent with recent publications on IMPC [6, 21]. In summary, while the heightened N-cad expression observed in the IMPC component within our cohort supports its metastatic potential, breast cancer cases within this group do not exhibit a shorter expected survival compared to those with IBC-NST. These findings align with the existing literature [6, 7, 14, 16, 20, 21]. Thus, we showed that the increased expression of CD44s and N-cad, which has been shown in basal-like or high-grade breast cancer [25, 28], is not present in IMPC.

We observed a significant reduction in membranous *β*-cat staining within the LNM, which correlated with a shortened DMFS in our investigation, as illustrated in Table 1. It is worth noting that we could not detect cytoplasmic and nuclear staining using this specific marker, a phenomenon consistent with prior studies [31, 32]. For instance, Cai et al. previously demonstrated that suppressing *β*-cat expression in human breast cancer MDA-MB-231 cells promoted EMT [30]. Interestingly, our investigation revealed no significant disparity in *β*-cat and E-cad expression levels between the IMPC and IBC-NST regions. However, a notable distinction emerged in the lymph node tumor area (*p* = 0.007), as depicted in Table 2. This discrepancy suggests that cases with reduced *β*-cat expression and a shorter DFS exhibit heightened E-cad expression within the metastatic lymph node. This incongruity in the metastatic setting might imply that *β*-cat acquisition occurs subsequent to E-cad acquisition in the tumor population having completed the EMT cycle, or it could indicate a lack of direct correlation between these two molecules within this context. In essence, this subset of tumors may still possess an inherent propensity for distant organ metastasis.

We observed noteworthy variations in expression intensities within the same patients and across tumor tissues in three distinct regions (Table 2) in the associations among EMT markers. Notably, our findings revealed a consistent upregulation of N-cad and CD44 expression across all three tumor tissue regions in conjunction with reduced *β*-cat membranous staining, and these differences were statistically significant. When examining the connection between *β*-cat and E-cad, we identified a disparity in their expressions solely within the lymph node region, while no such distinctions were evident in the other areas. This observation aligns with previous research, such as that by Ebert et al., who reported a parallel reduction in *β*-cat levels in metastatic gastric cancer cells, which they attributed to the *β*-cat promoter hypermethylation [32]. These collective findings substantiate the prevailing notion that E-cad and *β*-cat often function in concert [31, 33].

In our investigation, we observed a significant difference in the reduction of E-cad expression in tumor tissues, coinciding with an increase in N-cad expression, as presented in Table 2. This outcome aligns with the existing literature [26, 27] and substantiates the anticipated variance between these two molecules.

When examining the association between N-cad and CD44 expression, we identified distinct patterns in cases within the IBC-NST area, maintaining their stromal connections. Specifically, in instances where CD44 expression decreased, N-cad expression decreased, signifying a concordance as delineated in Table 2. Moreover, such cases were associated with a DFS period (Table 1). Conversely, within tumor regions representing the IMPC component and LNM, we observed an increase in N-cad expression when CD44 expression decreased, resulting in a disparity. These findings align with Nagi et al.'s study, which demonstrated higher N-cad expression in IMPC compared to IBC-NST [27], supporting the notion that these two molecules exhibit distinct relationships within the IMPC and IBC-NST contexts. Fundamentally, our observations suggest that cell groups in the IMPC area have severed their connections with the stromal microenvironment, potentially indicating divergent signaling pathways for the CD44 molecule's impact on tumor cells in the IBC-NST area, where stromal interactions persist, and the IMPC area. Alternatively, these findings may be construed as a manifestation of the dual effects of the CD44 molecule [42]. This result aligns with the conclusions of Kramer et al., who also found no significant difference in terms of DMFS between IMPC and IBC-NST cases while investigating EMT-related genes [19].

In summary, the outcomes of our study on IMPC do not provide support for the anticipated upregulation of CD44s, *β*-cat molecule displacement, and the loss of E-cad expression in IMPC cells, as expected in the classical EMT process. It is evident from our study that a decreased expression of CD44 has a prognostic impact on IMPC. Consequently, it appears that a therapeutic strategy targeting CD44 may not be suitable for IMPC as a stem cell biomarker. Nevertheless, the inclusion of EMT-related markers in routine assessments may offer valuable perspectives and prognostic insights in such cases that do not even have LNM. In the context of IMPC, which exhibits limited communication with the ECM, we can hypothesize that the loss of CD44s in morule-like tumoral groups is a natural process, as the CD44 molecule might not receive ligand signals. Additionally, our study observed a moderate reduction in E-cad expression in these cells. To ascertain whether the loss of CD44 is a mechanism triggering the formation of small cell clusters, as observed in IMPC, further in vitro experiments and longitudinal studies are warranted. Conversely, it is noteworthy that increased CD44s expression has been documented in high-grade breast tumors, with purported associations with N-cad [2].

Consequently, we can assert that EMT process does not appear to exacerbate metastasis in IMPC. Notably, our IMPC research cohort primarily comprises luminal molecular subtypes. The outcomes of our molecular classification and our investigations into EMT molecules collectively challenge the classification of mixed IMPC as belonging to the high-grade, poor prognostic breast tumor category, which has been suggested by previous studies [7, 12, 14, 16, 20, 21]. Additionally, we have investigated concurrently the distinct expression profiles and interconnections of crucial molecules involved in the initiation of the EMT process within both the primary tumor mass, specifically IMPC and IBC-NST, as well as the associated LNM regions for the first time in the literature. This conclusion, drawn from our molecular and prognostic data, aligns with recent investigations into the prognostic attributes of IMPC cases, albeit in contrast to some earlier studies and reports [6, 15, 17, 18, 38, 45].

Given the absence of significant molecular distinctions between IMPC and IBC-NST domains, our findings are consistent with contemporary literature, suggesting that the prognosis of IMPC may not be inherently worse than that of IBC-NST [12, 13, 19, 21, 22]. Our study has yielded a wealth of data that helps elucidate the behavioral patterns of IMPC. The observation of analogous molecular expression profiles in both IMPC and IBC-NST regions, particularly within the same case, aligns harmoniously with recent scientific discourse. Consequently, it is our conclusion that in-depth, genetically based multiomic studies should be deemed imperative to advance our understanding of this intriguing subject further.

## Figures and Tables

**Table 1:** The number of cases according to prognostic biomarkers staining and their relationship with overall survival time and disease-free survival time (months).

	**Number of the cases**	**Overall survival (months)**	**p** **value**	**Disease-free survival (months)**	**p** **value**
CD44 I expression					
Negative (scores 0–6)	24 (38.70%)	116.142 ± 20.040		134.80 ± 26.10	
Positive (scores 7–9)	38 (61.30%)	154.506 ± 11.684	0.010	139.19 ± 13.10	0.420
CD44 II expression					
Negative (score 0–6)	18 (29.03%)	67.981 ± 9.027		59.238 ± 9.931	
Positive (score 7–9)	44 (70.97%)	182.627 ± 15.179	<0.0005	181.447 ± 15.556	0.005
CD44 III expression					
Negative (scores 0–6)	20 (32.25%)	99.971 ± 14.779		104.05 ± 18.40	
Positive (score 7–9)	42 (67.75%)	179.648 ± 15.907	0.025	169.83 ± 16.99	0.410
N-cad I expression					
Negative (scores 0–3)	33 (53.22%)	172.08 ± 17.91		186.591 ± 17.705	
Positive (scores 4–9)	29 (46.88%)	118.17 ± 14.78	0.181	105.904 ± 16.189	0.041
*β*-cat III expression					
Negative (scores 0–3)	10 (16.12%)	121.87 ± 31.20		90.222 ± 30.162	
Positive (scores 4–9)	52 (83.88%)	136.20 ± 10.28	0.183	140.883 ± 10.851	0.009

**Table 2:** *p* values (McNemar's test) showing the inconsistency/consistency of the staining intensity of MPK (I), second component (II), and lymph node metastatic (III) tumor areas within itself and with other markers.

	***β*** **-cat I**	***β*** **-cat II**	***β*** **-cat III**	**N-cad I**	**N-cad II**	**N-cad III**	**CD44 I**	**CD44 II**	**CD44 II**
N-cad I	<0.000								
N-cad II		<0.000							
N-cad III			<0.000						
CD44 I	0.021			0.015					
CD44 II		0.005			0.233				
CD44 III			0.012			0.001			
E-cad I	0.180			<0.000			0.345		
E-cad II		0.167			<0.000			0.980	
E-cad III			0.007			<0.000			0.701

## Data Availability

The radiology, laboratory, and pathology data used to support the findings of this study can be accessed by applying to the local ethics committee of Health Sciences Research of Health Sciences University Izmir Bozyaka Training and Research Hospital.

## Abbreviations

IMPC: Invasive micropapillary carcinoma
LVI: Lymphovascular invasion
LNM: Lymph node metastasis
IHC: Immunohistochemical
IBC-NST: Invasive breast carcinoma, no-special type
EMT: Epithelial–mesenchymal transition
OS: Overall survival
DFS: Disease-free survival
DEUMF: Dokuz Eylul University Medical Faculty
UHSIBTRH: University of Health Sciences Izmir Bozyaka Training and Research Hospital
ASCO/CAP: American Society of Clinical Oncology/College of American Pathologists.
