# Supplementary material for: Comprehensive Immunohistochemical Analysis of Epithelial–Mesenchymal Transition Biomarkers in the Invasive Micropapillary Cancer of the Breast
Source: Int J Breast Cancer. 2024 Jun 11;2024:2350073. doi: 10.1155/2024/2350073 (PMC11189676; doi:10.1155/2024/2350073)
Supplement: Supporting Information — Additional supporting information can be found online in the Supporting Information section. Immunohistochemical stains used in the study are listed in Table S1. [file 2350073.f1.docx]

| Supplementary Tabel S1. Immunohistochemical Stains Used in the Study | | | |
| --- | --- | --- | --- |
| Antibodies | **Source** | **Clone** | **Dilution** |
| ER | Dako | EP1 | Ready to use (RTU) |
| PR | Dako | PgR 636, PgR 1294 | RTU |
| HER-2 | Dako | Polyclonal(A0485) | 1:1200 |
| Kİ-67 | Dako | MIB-1 | RTU |
| CD44s | Dako | DF1485 | 1/50 |
| E-cad | Dako | NCH-38 | RTU |
| N-cad | Santa Cruz | H-2 | 1/100 |
| B-cat | Dako | β-Catenin-1 | RTU |
